# Comparison of Two Methods of Capillary Sampling in Blood Pre-Donation Anemia Screening in Brazil

**DOI:** 10.3390/hematolrep15020030

**Published:** 2023-04-26

**Authors:** Cristina Rabelo Flor, André de Oliveira Baldoni, Sheila de Oliveira Garcia Mateos, Ester Cerdeira Sabino, Cláudia Di Lorenzo Oliveira

**Affiliations:** 1 Centro-Oeste Campus, Federal University of São João Del Rei (UFSJ), Divinópolis 35.501-296, MG, Brazil; andrebaldoni@ufsj.edu.br (A.d.O.B.); claudia.dlorenzo@gmail.com (C.D.L.O.); 2 Departamento de Moléstias Infecciosas, Parasitárias, Faculdade de Medicina Universidade de São Paulo, São Paulo 05.403-000, SP, Brazil; she.garcia@hotmail.com (S.d.O.G.M.); sabinoec@gmail.com (E.C.S.); 3 Hemorio Foundation, Rio de Janeiro 20.211-030, RJ, Brazil

**Keywords:** hemoglobin, hematocrit, agreement, anemia

## Abstract

Background: The laboratory tests most used by blood banks to diagnose anemia are the hemoglobin (Hb) and microhematocrit (Hct) tests, measured from capillary samples. Objective: To analyze the two capillary screening methods for pre-donation anemia by comparing their agreement in diagnosing anemia. Method: A cross-sectional study in a population of 15,521 blood donation candidates for whom information was available on Hb and Hct, performed from capillary blood samples. Hb was determined using the HemoCue^®^ test and Hct by the centrifugation method. The Kappa coefficient was calculated to assess the agreement between the methods. Pearson’s correlation tests and gender-adjusted linear regression were used to assess the change in the response variable (Hb) as a function of the explanatory variable (Hct). Results: The majority of the study population were men (70.4%), aged between 18 and 44 years (72.1%), who declared themselves white or mixed skin color (85.6%), and had undergone at least 11 years of complete education (72.4%). The Kappa coefficient found was 92.7 and 99.2 for women and men, respectively. Pearson’s correlation showed a correlation coefficient of 0.98 and the linear regression graph showed an adequate relationship between the tests with R^2^ = 0.97. Conclusions: Comparing the Hb and Hct capillary tests, it was found that Hct can be safely used to screen for anemia in pre-blood donation.

## 1. Introduction

Blood transfusion is an irreplaceable therapeutic procedure for various clinical conditions, such as volume replacement in surgeries and trauma, in the treatment of severe anemia and severe hematological diseases, such as thalassemia and hemophilia [1,2]. To minimize the risks of the procedure, the quality and safety of blood and its components must be guaranteed, from the selection of donors to the correct indication, prescription, and administration of the product to the patient [2].

The selection of blood donors is critical for the safety of blood transfusion. Blood donor eligibility policies aim to standardize practices and stipulate essential criteria to ensure protection and well-being for the blood donor, and safety for the recipient of this blood and its components [3]. Anemia is one of the most common causes of blood being unfit for donation, especially among women [4,5,6]. In addition, the donor has a greater chance of developing iron depletion anemia, since this condition has been strongly associated with repeat blood donations and with shorter intervals between donations [7,8].

Anemia is a physiopathologically diverse disease and has a multifactorial cause characterized by a decrease in red blood cells in the blood, accompanied by decreased levels of hemoglobin or changes in the morphology of red blood cells, which can compromise cellular oxygenation [9,10]. The gold standard diagnosis of anemia is usually made by the level of hemoglobin (Hb), since this parameter provides a direct measure of the oxygen carrying capacity in the blood. However, microhematocrit (Hct) can serve as an indirect parameter for this purpose [11], in addition to being a method with better economic viability [12]. The criteria for choosing the appropriate pre-donation anemia screening method should be based on its accuracy, speed, operability, and minimal discomfort to the blood donor [12].

Despite the Brazilian Ministry of Health 05/2017 ordinance [13] which recommends both Hb and/or Hct capillary tests for anemia screening in blood donation candidates, there are few studies comparing these tests [14,15] and none regarding blood donors. Therefore, this article aims to compare two methods of screening for pre-donation anemia (Hb and micro-Hct) using a capillary sample as to the extent of their agreement in the diagnosis of anemia.

## 2. Method

### 2.1. Study Design

A retrospective cross-sectional study [14] was conducted on a population of blood donation candidates who attended the Hemorio Foundation between 2006 and 2012. All donations that met the inclusion criteria were included in the study. The inclusion criteria were to have both laboratory tests (Hb and Hct). Data were obtained from the multicenter Retrovirus Epidemiology Donor Study II (REDS II) research program. REDS II is a multicentric research program funded and financed by the National Institute of Health (NIH) of the United States of America, which included Brazil as an international component in 2006. The main objectives of this program were to assess transfusion safety and the adequacy of blood supply in Brazil [15,16].

### 2.2. Study Population

The study population consisted of candidates for blood donation from the Hemorio Foundation, Rio de Janeiro, Brazil. The data were obtained from the clinical and sociodemographic history collected, and laboratory tests in the routine care performed by this blood bank. Blood donors and blood donation candidates for whom information was available on Hb and Hct values made from capillary samples were included in the study.

### 2.3. Laboratory Tests

Quantification of Hb was obtained using the HemoCue^®^ test and Hct was performed by centrifugation.

Around 10 μL of whole blood obtained through the fingertip puncture procedure was used directly in a standard capillary tube for microhematocrit centrifuges to obtain hematocrit values, and the result was read manually after 1 min of centrifugation.

A drop of whole blood (10 μL) was used and analyzed on the Hemocue equipment to determine the hemoglobin value, using a disposable micro cuvette that allows for greater safety in the procedure. The equipment’s basic concept is red blood cell lysis, and the hemoglobin is released and converted to azide-methemoglobin and subsequently read at 570 nm and 880 nm by spectrophotometry to account for the turbidity. The result was obtained between 15 and 60 s, read directly on the display of the equipment and recorded in the blood donor system. According to the manufacturer’s instructions, quality control was carried out through an internal electronic self-test program.

### 2.4. Statistical Analysis

The descriptive analysis of the participants was carried out by frequency distribution according to sociodemographic variables. The study participants were classified as anemic or non-anemic, based on the reference values of Hb and Hct defined by Ordinance 05/2017 of the Brazilian Ministry of Health [13]. The minimum acceptable values of Hb and Hct for blood donation are 12.5 g/dL and 38%, and 13 g/dL and 39% for women and men respectively [13]. The measures of central tendency of Hb and Hct values and the variation of their results for men and women were calculated.

We calculated the Kappa coefficient, used for assessment of agreement or reliability between two measures, to compare Hct and Hb, both using capillary samples. Pearson’s correlation test was used to validate the correlation between Hb and Hct. Linear regression adjusted by gender was used to assess the change in the response variable (Hb) as a function of the explanatory variable (Hct), since Hb is considered the gold standard for anemia screening. Statistical analyses were performed using STATA 12.0^®^ software.

## 3. Results

The study population consisted of 15,521 blood donation candidates who attended Hemorio between 2006 and 2012 and had Hb and Hct measured. Of these, the majority were men (70.4%), aged between 18 and 44 years (72.1%), declared themselves white or mixed skin color (85.6%), and had undergone at least 11 years of complete education (72.4%) (Table 1).

The Hb and Hct results of the blood donation candidates are summarized in Table 2. For both tests, mean and median were lower among women, a population that also showed a greater variation in the measured values. The ratio between the values of Hct and Hb (Hct/Hb) varied from 2.36 to 3.59, with mean and median equal to 2.94.

The percentage of women who presented anemia was higher compared to the men among those study participants who presented anemia (Table 3). The Kappa coefficient was 92.7 for women and 99.2 for men, and sensitivity and specificity of Hct in relation to Hb was 100% and >99% respectively for both genders equally.

**Figure 1:**
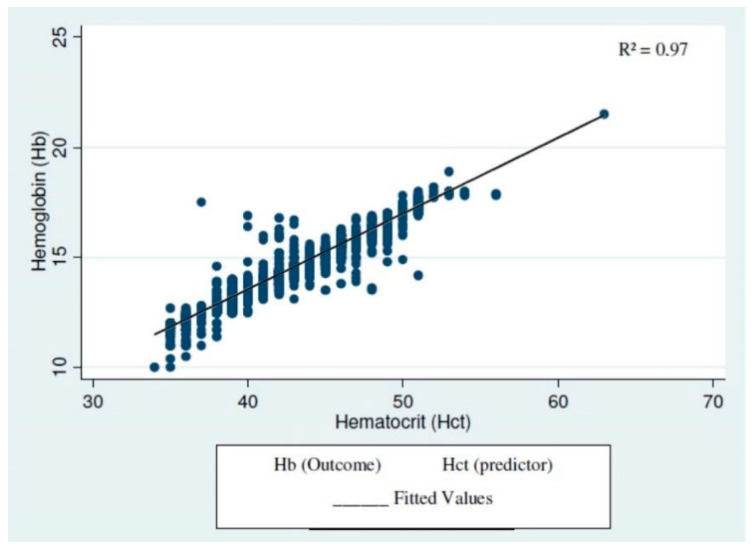
Linear regression between hemoglobin and hematocrit adjusted by gender (N = 15,521).

Figure 1 illustrates the linear relationship between Hb and Hct adjusted for gender, calculated using regression. There is clearly an adequate relationship between the tests and a good fit of the model. Pearson’s correlation showed a correlation coefficient of 0.98.

## 4. Discussion

Comparing the Hb and Hct capillary tests, we found that Hct can be safely used to screen for anemia in pre-blood donation. The Kappa coefficient showed a high degree of Hct in relation to Hb for both genders. Sensitivity and specificity values of Hct in relation to Hb were close to 100%; however, it should be noted that capillary Hb cannot be considered the gold standard in the diagnosis of anemia. In addition, a good statistical relationship was observed between the tests both in linear regression and in correlation.

Previous studies point to a greater agreement of capillary Hb in relation to Hct in blood donation candidates [11,12,17]. Most studies claim that both capillary Hb and Hct can be used to screen for anemia [12,17,18], despite their limitations. A study carried out with children concluded that Hb and Hct can be used together or separately to assess the prevalence of anemia, even with small differences depending on the cut-off point [17]. In another study carried out with women candidates for blood donation, the authors state that although both capillary tests have high rates of impediment to blood donation in potential non-anemic donors, Hb has greater discriminatory power in the detection of anemia in relation to Hct [12]. However, the authors consider the operational difficulties of using venous Hb and the higher cost of capillary Hb in relation to Hct in pre-donation screening [12]. Another study carried out with children also showed that Hb is more sensitive than Hct for the diagnosis of mild to moderate anemia. However, the authors suggest that reassessment of the Hct cutoff point for the diagnosis of anemia could make the tests more equivalent in accuracy [11]. Therefore, considering logistics and costs, we can say that capillary Hct is a reliable laboratory test for screening for pre-blood donation anemia.

Regarding the ratio between Hct and Hb (Hct/Hb), our study showed that this ratio can vary and that the consensus that Hct is equal to three times the value of Hb is inaccurate. Our findings corroborate previous studies that stated that although the Hb and Hct values are directly related, the Hct/Hb ratio is variable [11,19]. This inaccuracy is due to the influence of factors inherent in individuals that interfere with blood tests, such as dehydration or the presence of disease such as thalassemia [20]. A study carried out with children with malaria in endemic regions concluded that the conversion of Hb and Hct can also be influenced by variables such as gender and age [19]. However, the mean and median calculated in our study (2.94) coincide with the equation Hct = Hb × 2.941, proposed in a study on the accuracy of a method for measuring Hct and estimating Hb [21].

Our study has limitations. The first of these is the absence of venous blood for comparison with a capillary sample, since the collection of venous blood is not performed in the screening and selection of donors in the routine of services at the blood bank. The second limitation is that the tests were not performed for research and comparison purposes, and this can generate some variability in the collection technique between the different professionals who collected the blood. In contrast, it is important to emphasize that the blood bank’s technical and operational conduct protocols define rigid and standardized criteria for the performance of tests [22]. The third limitation of the study refers to its population, consisting of candidates for blood donation from a single blood bank, and is therefore not representative of the general population. However, our study had a large sample size and was representative of candidates of both genders, guaranteeing variability in the sample. In addition, this fact does not directly interfere with the analysis of agreement between the tests.

## 5. Conclusions

Since anemia is one of the main causes of impediment to blood donation, the performance of diagnostic tests that identify it precisely in the process of verifying suitability for donation, at an affordable cost, is very important in ensuring the safety of the donor and the recipient of the blood. Comparing the Hb and Hct tests obtained by capillary sampling, we found that Hct can be used safely to screen for anemia in pre-blood donation. In order to evaluate these results for application in public health, we observed the necessity for new studies comparing tests in populations with different risks of anemia: women in reproductive age, elderly, and children.

## Figures and Tables

**Table 1:** Sociodemographic characteristics of the study population (N = 15,521).

Variable	Frequency (%)
*Gender*	
Female	4598 (29.6%)
Male	10,923 (70.4%)
*Age* (*years*)	36.51 ± 9.13
18 to 24 years	2526 (16.3%)
25 to 34 years	4839 (31.2%)
35 to 44 years	3824 (24.6%)
45 to 54 years	2633 (17.0%)
55 to 65 years	985 (6.3%)
No information	714 (4.6%)
*Self-declared skin color*	
Mixed	6306 (40.6%)
White	6985 (45.0%)
Black	1261 (8.1%)
Other	72 (0.5%)
No response	897 (5.8%)
*Education*	
Less than 8 years of schooling	2470 (16.0%)
8 to 10 years of schooling	1806 (11.6%)
11 years of schooling	7750 (49.9%)
Graduation or above	3495 (22.5%)

**Table 2:** Description of the hemoglobin and hematocrit results of the study participants stratified by gender (N = 15,521).

	Gender			
	Female (N = 4598)		Male (N = 10,923)	
	Hb (g/dL)	Hct (%)	Hb (g/dL)	Hct (%)
Mean ± SD	13.5 ± 0.8	39.9 ± 2.3	14.9 ± 1.1	43.8 ± 3.2
Median	13.4	39.0	14.8	44.0
Range	10.0–21.5	34–63	11.0–18.9	35–56

**Table 3:** Screening for anemia by hemoglobin and hematocrit levels of study participants by gender (N = 15,521).

Hematological Variables	Gender			
	Female * (N = 4598)		Male ** (N = 10,923)	
	Anemia	Non Anemia	Anemia	Non Anemia
Hemoglobin ***	246 (5.4%)	4352 (94.6%)	126 (1.2%)	10,797 (98.8%)
Hematocrit ****	282 (6.1%)	4316 (93.9%)	128 (1.2%)	10,795 (98.8%)

* Women: Kappa coefficient = 92.7. ** Men: Kappa coefficient = 99.2. *** Women with Hb < 12.5 g/dL and men with Hb < 13.0 g/dL were considered with anemia. **** women with Hct < 38% and men with Hct < 39% were considered with anemia.

## Data Availability

Data sharing not applicable. No new data were created or analyzed in this study. Data sharing is not applicable to this article.
